# Pathology and virology of natural high pathogenicity avian influenza A(H5N1) Gs/GD genotype BB virus infection in wild black-headed gulls (*Chroicocephalus ridibundus*)

**DOI:** 10.1186/s13567-025-01666-x

**Published:** 2025-12-29

**Authors:** Edwin J. B. Veldhuis Kroeze, Beatriz Bellido Martin, Vera C. Mols, Lineke Begeman, Ron A. M. Fouchier, Thijs Kuiken

**Affiliations:** Department of Viroscience, Erasmus University Medical Centre, Rotterdam, The Netherlands

**Keywords:** HPAI H5N1 virus, Clade 2.3.4.4b, BB genotype, pathogenesis, histopathology, virology, epidemiology, outbreak mass mortality, colony-breeding black-headed gull, common tern

## Abstract

The ongoing worldwide outbreak of high pathogenicity avian influenza (HPAI) H5 of the Goose/Guangdong (Gs/GD) lineage has caused unprecedented mortality amongst an ever-expanding number of avian and mammalian species. This includes black-headed gulls, which suffered high mortality from this virus in 2022 and 2023. We performed pathological and virological analyses on black-headed gulls and a common tern found dead in a breeding site in The Netherlands in spring 2023 in order to characterise the pathogenesis of HPAI Gs/GD virus infection in these species. We found a strong tropism of HPAI H5 Gs/GD virus for the central nervous system, pancreas, lungs, and the preen gland, with acute necrosis as the main associated lesion. Affected organs revealed high virus RNA loads and intralesional expression of virus antigen, confirming HPAI H5 Gs/GD virus infection as the cause of disease and mortality. Genetic sequencing showed that this HPAI H5 Gs/GD clade 2.3.4.4b virus belonged to the gull-adapted BB genotype. Comprehensive pathogenesis studies in a cohort of wild animals naturally infected with HPAI H5 Gs/GD virus are scarce, yet the knowledge gained may fill knowledge gaps to help mitigate future HPAI outbreaks.

**Supplementary Information:**

The online version contains supplementary material available at 10.1186/s13567-025-01666-x.

## Introduction

The ongoing worldwide outbreak of high pathogenicity avian influenza (HPAI) H5 virus of the Goose/Guangdong (Gs/GD) lineage has affected a broad range of avian and mammalian species, including humans, and in some species has caused high mortality. So far, HPAI H5 Gs/GD viruses have been reported in at least 525 species of wild birds and 80 species of non-human mammals [1]. The cumulative mortality caused by HPAI H5 Gs/GD viruses includes more than 400 million chickens and other poultry [2] and about 300 000 fur-bearing mammals in Finland [3] and Spain [4], as well as 530 people out of 1080 with confirmed infection [1]. International reporting on the level of mortality from HPAI H5 Gs/GD viruses in free-living wildlife is severely lacking [5], but there are well-documented examples showing high mortality in both wild birds [6] and wild mammals [7].

Black-headed gulls (*Chroicocephalus ridibundus*) are one of the wild bird species that have suffered high mortality from HPAI H5 Gs/GD virus. Starting in autumn 2022, and throughout 2023, high mortality of black-headed gulls from HPAI H5 Gs/GD virus infection was recorded at wintering sites in Italy, France, and The Netherlands, and at colony breeding sites in Belgium, Czechia, Germany, The Netherlands, Poland, Sweden, and the United Kingdom [8–11]. This high and widespread mortality is of concern for black-headed gulls, both in terms of loss of individual lives and reduction of their population numbers.

HPAI H5 Gs/GD virus was first detected in 1996 in a commercial farm of domestic geese in Guangdong Province, China [12]. Like other HPAI viruses, the emergence of Gs/GD H5 virus was likely due to mutation from a low pathogenicity avian influenza (LPAI) virus [13]. After spreading regionally in poultry in Asia in the first years after its emergence, HPAI H5 Gs/GD virus spilled over significantly into wild birds in subsequent years, allowing long-distance spread via wild bird migration to other continents, including Europe [14]. It was shown by phylogenetic analysis that the 2.3.4.4b clade of HPAI H5 Gs/GD virus was able to persist in wild bird populations in Europe from 2020 onwards [15]. Although black-headed gulls had exhibited sporadic mortality from HPAI H5 Gs/GD virus before [16, 17] high and widespread mortality only started when they became infected with genotype BB (H5N1 A/Herring gull/France/22P015977/2022-like). This virus was a reassortant of HPAI H5 Gs/GD virus and a gull-adapted LPAIV H13 virus (from which it acquired the NP, PA and NS genes), and was first detected in a herring gull in France in May 2022 [18].

The black-headed gull breeds across Europe and Asia between the latitudes of about 40 and 70 degrees north, with small breeding populations in Iceland, Greenland and the east coast of North America. The global population of black-headed gulls is about 2–3 million breeding pairs. Black-headed gulls breed in dense colonies often of several thousands, but rarely > 10 000 pairs, mostly in lakes surrounded by reed beds with small islands; also coastal sand islands, bogs, and artificial ponds. In Europe, black-headed gulls breed in most countries, with higher numbers in the north [19].

The pathogenesis of avian influenza in black-headed gulls differs substantially between LPAI and HPAI. Natural infection with LPAI virus H16N3 or H13N8 was restricted to intestinal and bursal epithelium in the absence of lesions or clinical signs [20]. In contrast, experimental infection with HPAI H5N1 Gs/GD virus (A/turkey/Turkey/1/2005) caused high morbidity and mortality, spread to multiple organs including the respiratory tract, brain, heart and pancreas, but not intestine. There was virus antigen expression in parenchymal cells of infected organs, that was associated with necrosis and infiltration with variable proportions of heterophils, macrophages, lymphocytes and plasma cells [21].

The pathogenesis in black-headed gulls infected with HPAI H5N1 Gs/GD genotype BB virus, arising about two decades after HPAI H5N1 Gs/GD virus (A/turkey/Turkey/1/2005), is not known. Therefore, the goal of our study was to characterise the cell type tropism and associated lesions in black-headed gulls that were fatally infected with this virus, and to use this information to improve understanding of its pathogenesis. To do so, we performed virological and pathological analyses on carcasses of eleven black-headed gulls and one common tern (*Sterna hirundo*) that were naturally infected with HPAI H5N1 Gs/GD genotype BB virus. These carcasses were obtained from a die-off at a colony breeding site in The Netherlands early June 2023.

## Materials and methods

### Animals and breeding colonies

In the 2023 breeding season, a significant die-off amongst wild black-headed gulls was noted at a colony breeding site on a small man-made island called “de Kreupel” in the IIsselmeer, The Netherlands, during the outbreak of HPAI H5Nx (Gs/GD) in wildlife that has been ongoing since 2020. De Kreupel is an island group or archipelago composed of several small islands (Additional file 1) prohibited to public access. On June 2^nd^ 2023, twelve adult black-headed gulls and one common tern were collected from this site by permitted scientists. Because simultaneously and intermingled breeding terns were seen starting dying, the tern was included ad hoc in the study to compare results. Ten gulls were found freshly dead, and two gulls (gull-1 and 8) and the tern were found alive, moribund and unresponsive to stimuli, and were euthanised by cervical dislocation. Swabs for virology were taken from the oropharynx, cloaca, and preen gland from each bird in the field, using sterile cotton swabs that were subsequently each stored in 1.0 mL of virus transport medium (VTM) until further analyses. The preen gland or uropygial gland is a bird-specific sebaceous gland in the dorsal midline near the tail base, and that exudes waxy lipids used by birds to waterproof plumage. Swabs were taken from the excretory orifice of the glands to evaluate possible virus excretion.

We planned to test the island’s wetlands and waters for possible virus contamination. Contrary to the islands where the birds were collected, only the southernmost island of the archipelago (Additional file 1) held shallow stagnant rainwater pools. Whilst sampling the three pools (W4-6, 2 mL each), used by gulls and terns, the water surrounding this southernmost island was sampled on five locations also (W1-3, W7, W8, 2 mL each) (Additional file 2). Bird carcasses were scattered throughout the archipelago’s islands and waters. The water samples were analysed, without pre-concentration by centrifugal filters to optimise virus yield as described by Stallknecht et al. 2010 [22], by rRT-PCR for possible virus RNA presence and loads.

The bird carcasses and water samples were individually placed in sealed plastic biosafety bags and kept on wet ice until autopsies and further analyses were performed at the Erasmus University Medical Centre Rotterdam under Biosecurity-Level-3 conditions on the next day within 24 h after retrieval.

### Autopsies

Autopsies and tissue sampling were carried out according to a standard protocol. From each bird, samples from brain, lung, liver, spleen, kidney, heart, blood clots from the heart, pancreas, colon and feather shafts (proximal 1.5 cm of the first three secondary wing feathers per bird) were collected into empty ampoules, capped and stored at 4 ℃ for ≤ 3 h until virological analysis. Duplicate samples of the previously listed tissues (excluding the blood clots), as well as samples of spinal cord, eye, trachea, air sacs, proventriculus, small intestine, gonads, and preen gland were fixed in 10% neutral-buffered formalin at room temperature until histopathological analysis.

### Virology

Tissue samples were homogenised with a FastPrep 24 (MP Biomedicals, Eindhoven, The Netherlands) in Hankʾs balanced salt solution and centrifuged briefly before dilution in lysis buffer for RNA extraction. RNA extraction from all tissue samples, and from oropharyngeal, cloacal and preen gland swabs was performed on the MagNA Pure 96 platform (Roche Diagnostics GmbH, Mannheim, Germany). Phocine distemper virus (PDV) was added and used as internal control. Subsequently, all samples were tested in a triplex rRT-PCR targeting the matrix gene, haemagglutinin H5 and PDV [23]. The resulting Ct-values give an indication of the influenza A virus RNA load in each sample, lower values equal higher loads, and vice versa.

To determine the whole genome consensus sequence of HPAI H5 viruses, RNA was re-extracted from brain tissue and oropharyngeal swab from gull-11 and from oropharyngeal swab from the tern, using the High Pure RNA Isolation Kit (Roche Diagnostics GmbH Mannheim). A multi-segment RT-PCR amplification was performed using the Superscript III high-fidelity RT-PCR Kit (Invitro-gen, USA). Influenza virus specific primers were used, containing 13 conserved nucleotides at the 5’ terminus and 12 nucleotides and unique barcoded primers at the 3’ terminus, covering all eight influenza gene segments [23]. The libraries were generated using a ligation sequencing kit (SQK-LSK114, Oxford Nanopore technologies) and multiplexed and sequenced on a MinION R10 flowcell (Oxford Nanopore technologies) according to the manufacturer’s instructions. Porechop software was used to demultiplex the reads that contained a barcode. For analysis, FASTQ-files were imported to the CLC Genomics Workbench v20.0.03 (QIAGEN) and analyzed as described previously [23]. Sequences were mapped to reference sequences of H1-H16 and N1-N9 subtypes available on GISAID [24]. To extract the consensus genomes the following parameters were used: match score = 1, mismatch cost = 2, length fraction = 0.7, and similarity fraction = 0.8. Genome sequences were deposited and available from GISAID database [25] with accession numbers: EPI_ISL_19854913 (A/Black-headed gull/Netherlands/125-1/2023; brain), EPI_ISL_19855567 (A/Black-headed gull/Netherlands/125-2/2023; oropharyngeal swab), and EPI_ISL_19857471 (A/Common tern/Netherlands/44-2/2023; oropharyngeal swab).

### Histopathology and immunohistochemistry

The formalin-fixed tissues samples were processed routinely: embedded in paraffin, sectioned at 4 μm and stained with hematoxylin and eosin (H&E); serial sections were stained by immunohistochemistry (IHC) using AEC-immuno-peroxidase in conjunction with a monoclonal antibody against influenza A virus nucleoprotein (NP); a mouse IgG2a-anti-Influenza A NP (Clone Hb65, American Type Culture Collection) on a hematoxylin counterstain [26]. All H&E-stained slides were evaluated by light microscopy for any histological lesions, while IHC-stained slides were evaluated for virus NP expression.

## Results

### Autopsy

Upon autopsy, none of the birds showed external lesions. All were in a relatively good state of preservation. Four gulls and the one tern were females evidenced by the intra-coelomic presence of an oviduct and ovary, and seven gulls were males evidenced by the presence of testes. All birds were breeding adults evidenced by their well-developed gonads and their breeding plumage. Their nutritional state ranged from poor to good, but was moderate in most (7 of 12; 58%) birds according to the presence of subcutaneous and coelomic fat reserves and to the development of pectoral musculature. Feed content was absent in most gastro-intestinal tracts. Only two gulls presented gross lesions. Gull-6 had several well-delimited petechial haemorrhages of approximately 1 mm in diameter on the periost of the skull. The spleen and liver of gull-9 appeared swollen, both with rounded edges.

### Virology

Virus RNA extraction and whole genome sequencing of brain tissue and/or oropharyngeal swabs from gull-11 and from the tern identified the virus as HPAI H5N1 Gs/GD clade 2.3.4.4b BB genotype. All gene segments were 99% to 100% identical to those of other known BB genotype viruses that circulated around this time in Europe available from GISAID [24].

Presenting Ct-values in the following result sections, the averages and ratios were calculated for 11 gulls instead of 12, because gull-2 was excluded after testing negative for virus RNA and negative for virus antigen expression in all swab and tissue samples (see also last paragraph of results). All other lungs tested influenza A(H5) virus RNA positive with an average Ct-value of 23 from the gulls, and Ct-value 21 from the tern. Most extra-respiratory tissue samples and blood clots tested RNA positive. Despite limited variation in averaged Ct-values (between parentheses), extra-respiratory gull tissues with higher virus RNA loads were: brain (18), pancreas (19), and spleen (24); and with relative lower loads were: liver (26), colon (26), blood clot (27), kidney (27), heart (28), and feather shaft (32). The Ct-values of the extra-respiratory tissues of the tern were in similar range (Table 1).

**Table 1:**
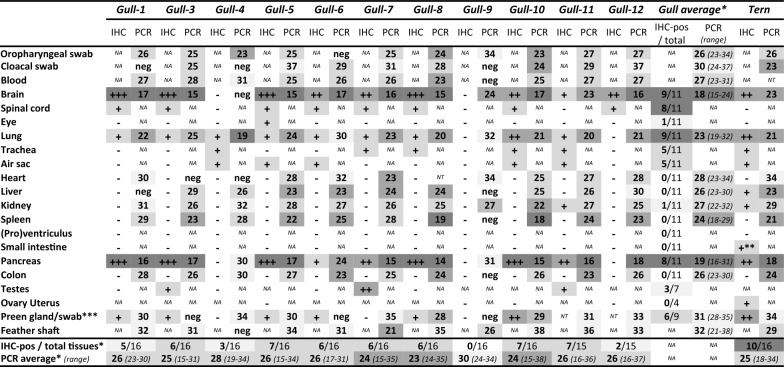
**Immunohistochemistry and rRT-PCR findings of HPAI H5N1 virus-infected black-headed gulls (*Chroicocephalus ridibundus*) and one common tern (*Sterna hirundo***)

Immunohistochemistry scores (IHC) and rRT-PCR values (PCR) for HPAIV H5N1-infected wild adult birds, eleven black-headed gulls, *Gull-1, 3* to *12*, and one common tern*. Gull-1* and *8*, and the *Tern* were euthanised, the others found dead. Data from *Gull-2* that tested negative in both IHC and rRT-PCR in all samples are not shown. IHC scores: −, no cells; + , low number of cells; +  + , medium number of cells; +  +  + , high number of cells showed positivity for influenza A viral nucleoprotein. rRT-PCR Cycle threshold (Ct) values of influenza A virus hemagglutinin H5 gene-fragment results from swabs, blood, and tissues with a cut-off value of ≥ 40 = negative (neg).

NA: not applicable, NT: not tested. Darker shaded values correspond with higher viral RNA loads and higher IHC scores, and vice versa.

^*^This column and the two bottom rows present the number of IHC positive tissues per total of tested tissues, and the average Ct-values of rRT-PCR positive samples, calculated for the gulls as group, and for each individual bird, respectively.

^**^IHC positive cells concerned only few neurons within the small intestinal Meissner’s submucosal nerve plexus of the tern (Figure 2C).

***rRT-PCR values determined from swabs of preen gland orifice.

Swabs taken from the oropharynx, cloaca, and preen gland tested positive for influenza A(H5) virus RNA in most birds. From the gulls, the oropharyngeal swabs tested more consistently positive with highest RNA loads (average Ct-value 26) compared to the cloacal swabs (average Ct-value 30) and preen gland swabs (average Ct-value 31). The tern showed similar results, although swabs from the cloaca yielded higher loads (Ct-value 23) than from the oropharynx (Ct-value 26) (Table 1). Additionally, one of eight water samples tested weakly influenza A(H5) virus RNA positive with a Ct-value of 39 just below the cut-off value of 40. This sample (W3) was taken from water that surrounds the southernmost island of the archipelago (Additional files 1 and 2A). The remaining water samples tested RNA negative (Additional files 1, 2B), possibly due to the sun’s UV-light or heating up [27], or both. The tested water samples were not concentrated to optimise virus yield as described previously [22].

### Histopathology and immunohistochemistry

**Figure 1:**
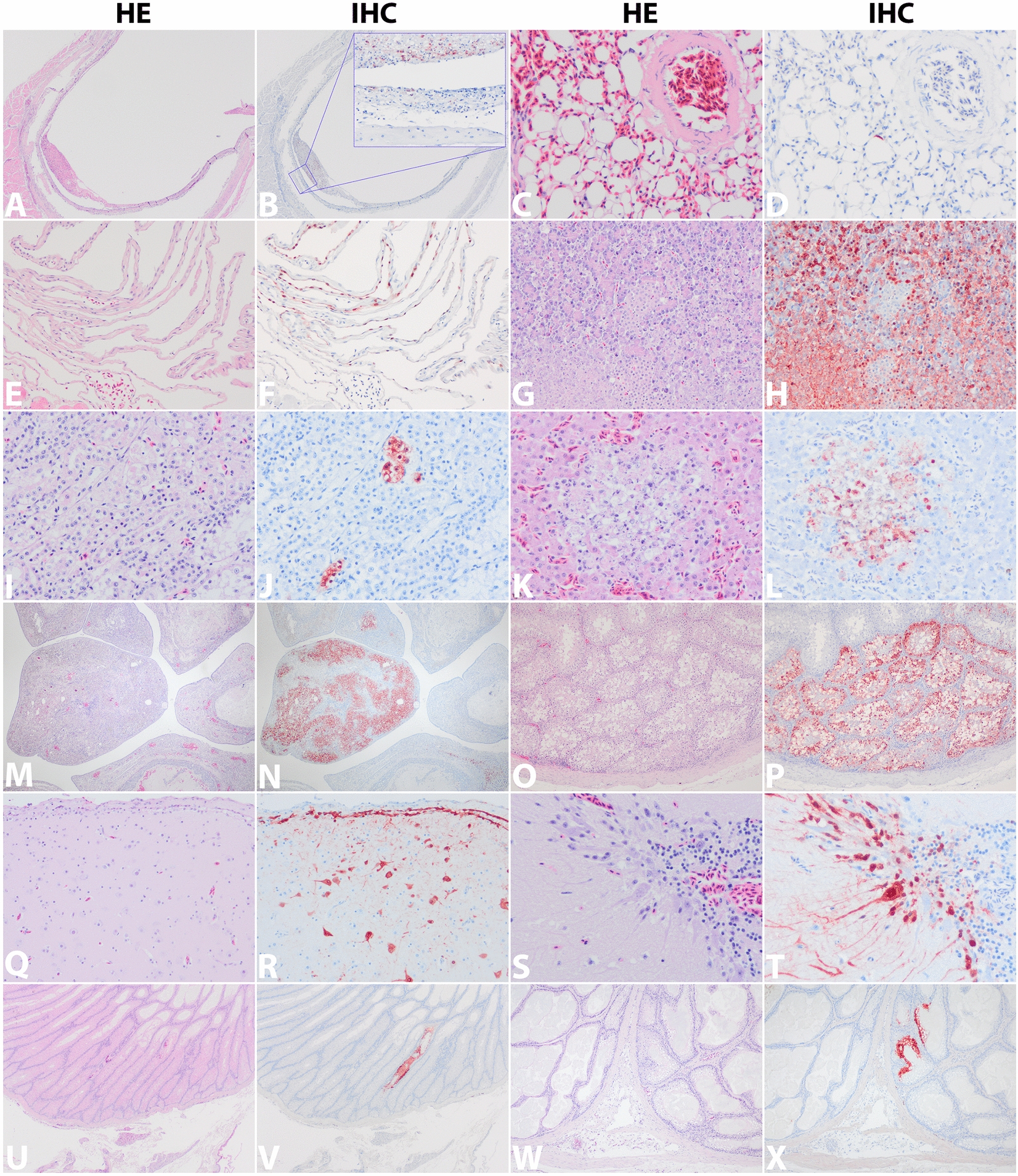
**Histopathology panel of organs of several black-headed gulls and a common tern in H&E staining (1**^**st**^ **and 3**^**rd**^ **column) in comparison with their respective serial sections presenting HPAIV H5N1 infection stained by IHC (2**^**nd**^ **and 3**^**rd**^ **column) in comparison with their respective serial sections presenting HPAIV H5N1 infection stained by IHC (2**^**nd**^ **and 4**^**th**^ **column)**; **A**, **B** cross section trachea with infected necrotic sloughed epithelial lining and exudate (20 × , gull-4); **C**, **D** hyperemic lungs with several infected alveolar capillary epithelial cell (200 × , gull-1); **E**, **F** air sac with infected epithelial lining cells (100 × , gull-4); **G**, **H** pancreas with marked necrosis of abundant infected exocrine glandular epithelial cells (100 × , gull-3); **I**, **J** kidney with foci of infected necrotic tubular epithelial cells (200 × , tern); **K**, **L** liver with foci of infected necrotic hepatocytes (200 × , tern); **M**, **N** ovary with focal abundance of infected and necrotic tubular and glandular epithelial cells (20 × , tern); **O**, **P** testis with infected necrotic seminiferous tubules (40 × , gull-7); **Q**, **R** cerebrum with infected cortical neurons and leptomeninges without inflammation (100 × , gull-1), **S**, **T** cerebellum with infected Purkinje cells especially, note positivity of axons (400 × , tern); **U**, **V**, **W**, **X** preen gland with infected degenerated to necrotic epithelial cells lining the glandular alveoli (20 × , gull-1, and 40 × , tern, respectively). Stained by IHC, positive influenza virus nucleoprotein (NP) antigen expression is visualised as finely granular reddish-brown staining by AEC-immuno-peroxidase on hematoxylin counterstain.

In general, severely affected organs and tissues expressed virus antigen especially within epithelial cells’ nuclei, that co-localised with necrosis. Within the respiratory system, air capillary epithelial cells in the lungs expressed virus antigen in low (8 of 11 gulls, 73%) to medium numbers of cells (1 of 11 gulls, 9%; and the tern). This virus antigen expression co-localised with pulmonary interstitial hyperemia and interstitial oedema, but without infiltration of inflammatory cells. Low numbers of epithelial cells lining the trachea and air sacs in 5 of 11 (45%) gulls and in the tern expressed virus antigen mostly devoid of inflammation, although the trachea of gull-4 expressed virus antigen in sloughed epithelial cells that co-localised with focal tracheal ulceration (Figure 1B).

**Figure 2:**
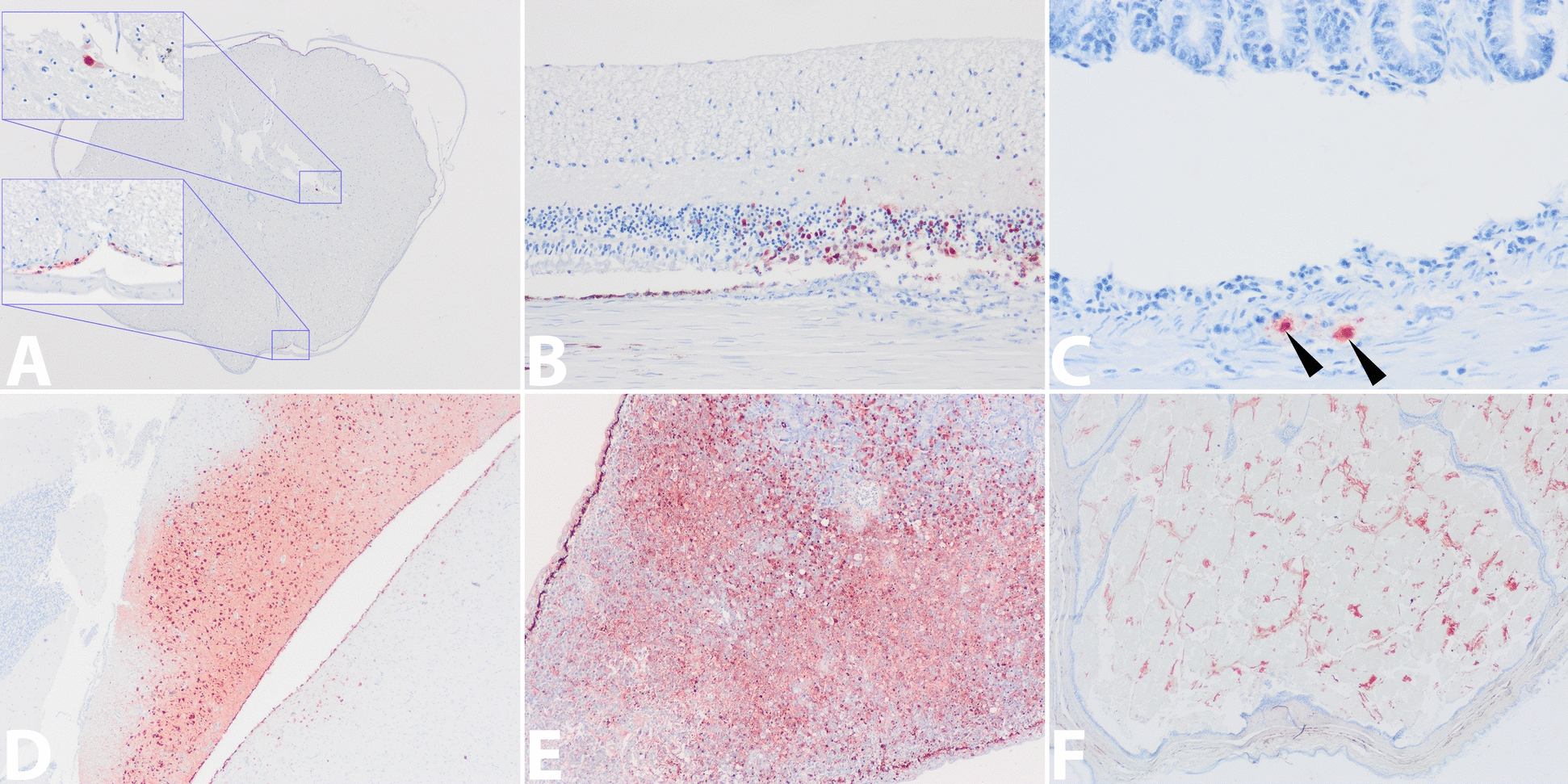
**Histopathology panel of specific organs of several black-headed gulls and a common tern presenting HPAI H5N1 virus’ tropism by IHC**; **A** cross section spinal cord motor neuron and leptomeningeal cells (20 × , inserts 200 × , gull-5); **B** retinal neurons (100 × , gull-5); **C** small intestinal submucosal Meissner’s nerve plexus neurons indicated by arrow heads (of note, the crypts at top detached by artefact from the submucosa on bottom, 100 × , tern); **D** cerebral neurons and ependymal cells lining the lateral ventricle (20 × , gull-1); **E** pancreatic exocrine glandular epithelial cells (40 × , gull-3); **F** preen gland epithelial cell debris and secretum (20 × , tern). Stained by IHC, positive influenza virus nucleoprotein (NP) antigen expression is visualised as finely granular reddish-brown staining by AEC-immuno-peroxidase on hematoxylin counterstain.

Within the nervous system, the brains of 9 of 11 (81%) of the gulls and the tern expressed virus antigen in medium to high numbers of neurons, glial cells, ependymal epithelial cells and in pial cells of the leptomeninges. Both nuclei and cytoplasm of neurons in the cerebrum (hippocampus), cerebellum (Purkinje cell layer) and brainstem expressed virus antigen. In some antigen-expressing neurons, there was acute necrosis evidenced by chromatolysis of the perikarya, pyknosis, karyolysis, cytoplasmic hypereosinophilia, or shrinking of neurons. Low numbers of neurons and leptomeningeal cells of the spinal cord in 8 of 11 (73%) of the gulls expressed virus antigen (Figure 2A). None of the brains and spinal cords showed infiltration with inflammatory cells or gliosis. A low number of neuronal cells of the retina in the eye of 1 of 11 (9%) gulls (Figure 2B), and two neuronal cells in the submucosal nerve plexus of Meissner in the small intestine of the tern, expressed virus antigen within the nucleus and cytoplam (Table 1 and Figure 2C).

Within the digestive system, no virus antigen was expressed in enterocytes lining the gastro-intestinal tract in any birds. High numbers of acinar epithelial cells of the exocrine pancreas expressed virus antigen, but not the endocrine cells within islets of Langerhans. Associated there was severe multifocal to coalescing pancreatic necrosis and oedema in 8 of 11 (73%) of the gulls and in the tern. Low numbers of hepatocytes in the liver (Figures 1K, L) of the tern only expressed virus antigen that co-localised with a few small foci of necrosis. Incidental parasite infections were observed in the intestines of four gulls. There were adult trematodes in the ilea of gull-1 and gull-2, thick-walled trematode ova in the duodenum of gull-3, and adult cestodes in the jejunum of gull-4.

Within other tissues, low numbers of tubular nephrocytes in the kidney (Figures 1I, J) of 1 of 11 (9%) gulls and the tern expressed virus antigen that co-localised with a few small foci of necrosis. Low numbers of tubular and glandular epithelial cells within the ovary of the tern, but none of the ovaries of the four female gulls, expressed virus antigen, whereas low to medium numbers of epithelial cells lining the seminiferous tubules of the testes in 3 of 7 (43%) of the male gulls expressed virus antigen. Virus antigen expression in these gonads co-localised with cellular necrosis and mild mixed cellular inflammation. Multifocally, a low to medium number of glandular epithelial cells of the preen gland in 6 of 9 (67%) of the gulls and the tern expressed virus antigen associated with necrosis without distinct inflammatory infiltrates. None of the other organs examined expressed virus antigen, necrosis or inflammation. Virus antigen expression was not observed in endothelial cells of any tissues.

Two gulls deserve separate mention. As gull-2 tested negative for virus RNA in all swabs and tissue samples, and tested negative for virus antigen expression in all tissue samples, and had no histological lesions, and because no alternative cause of death was apparent, its results were not included in calculating ratios and percentages and not presented in Table 1. Gull-9 tested positive for virus RNA in several key organs, but tested negative for virus antigen expression in all tissue samples, and had no histological lesions (Table 1). The tissues of this gull were more autolytic than those of the other gulls, and viral loads detected in these organs were relatively low, consequently the combination of autolysis and bacterial overgrowth may have hindered virus antigen expression.

## Discussion

We found virus-associated necrosis in multiple organs associated with the death of eleven free-living black-headed gulls and one common tern from a breeding site in The Netherlands that were naturally infected with HPAI H5N1 Gs/GD genotype BB virus early June, 2023. Below we discuss how this information adds to our knowledge of the pathogenesis of HPAI virus infection in black-headed gulls; for this purpose, we ask the following questions: what were the sites of primary virus replication, the routes of within-host virus spread and level of dissemination, cell type tropism in infected tissues, associated histological lesions, and sites of virus excretion?

Our findings suggest that the respiratory system was the site of initial virus replication. Supporting evidence is the high virus RNA load and marked virus antigen expression within the respiratory system. Similarly, virus antigen expression was observed in lung epithelial cells of two black-headed gulls found dead with HPAI H5N1 virus infection in England in 2022 [28], and in epithelial cells lining trachea, bronchi, and pulmonary air capillaries of black-headed gulls inoculated intratracheally and intraoesophageally with HPAI H5N1 Gs/GD virus (A/turkey/Turkey/1/2005) [21], but not in black-headed gulls naturally infected with LPAIV [20]. However, we cannot rule out the nervous system as (additional) site of initial virus replication, possibly by entry via cranial nerves such as olfactory nerves to the brain. This pathway was described for HPAI virus in mammals [29–31], yet conclusive evidence that HPAI virus uses this pathway to enter the brain in birds is lacking [32]. However, we can rule out the gastro-intestinal tract as site of initial virus replication, based on lack of virus antigen expression in epithelial cells lining proventriculus, ventriculus, small intestine and colon. Similar absence of virus antigen expression in gastro-intestinal epithelium was seen in a black-headed gull found dead in a zoo in France late 2022 infected with HPAI H5N1 Gs/GD genotype AB virus [33], and in black-headed gulls inoculated intratracheally and intraoesophageally with HPAI H5N1 Gs/GD virus (A/turkey/Turkey/1/2005) [21], but contrasts with black-headed gulls with natural LPAI H16N3 or H13N8 virus infection, that showed strong virus antigen expression in epithelial cells lining intestines at different levels [20].

Our findings suggest hematogenous spread of the virus to multiple extra-respiratory organs including the brain, and specific cell types within those organs. Supporting evidence are the extra-respiratory organs and blood found positive for virus RNA and antigen. However, vascular endothelial cells expressed no virus antigen in any of the tissues examined, which likely negates viral endothelial tropism. This contrasts with endotheliotropism observed in the proventriculus and gizzard of two black-headed gulls found dead with HPAI H5N1 virus infection in England in 2022 [28], but is consistent with absence of endotheliotropism in a black-headed gull found dead in a zoo in France late 2022 infected with HPAI H5N1 Gs/GD genotype AB virus [33], and in black-headed gulls inoculated experimentally with HPAI H5N1 Gs/GD virus (A/turkey/Turkey/1/2005) [21]. In general endotheliotropism of HPAIVs shows a species specific relationship [34, 35]. We cannot rule out (additional) contiguous spread from infected air sacs to organs in the coelomic cavity. Also we cannot rule out (additional) spread from infected brain to spinal cord (and to the plexus of Meissner of the intestine in the tern) via neurons. In black-headed gulls with natural LPAI H16N3 or H13N8 virus infection, no virus spread from virus-antigen-expressing epithelial cells lining the gastro-intestinal tract to neuronal cells of the submucosal nerve plexus nor any other organs was recorded [20].

Our findings suggest peracute or acute virus-associated necrosis of multiple organs resulting in rapid death, by which we mean that the birds died within several hours before the infected organs were infiltrated by inflammatory cells. Supporting evidence is the colocalisation of virus antigen expression and cellular necrosis in multiple organs, and absence of infiltration of heterophils and macrophages (characteristic of acute to subacute inflammation) or lymphocytes (characteristic of subacute to chronic inflammation). The most likely critically affected organ was the brain with neuronal necrosis, associated with neurological signs. Another organ severely affected by necrosis was the pancreas. This absence of inflammatory cell infiltration contrasts in particular with experimental infections of black-headed gulls with an older strain of HPAI H5N1 Gs/GD virus (A/turkey/Turkey/1/2005), where high numbers of inflammatory cells colocalised with virus antigen expression in brain and pancreas [21]. A possible explanation for the lack of inflammation is that the gulls under study here died so soon after infection that, despite severe necrosis, inflammatory cells had no time to reach the critically affected organs.

Our findings suggest the respiratory tract, digestive tract, urinary tract, reproductive tract, and preen gland as potential sites of virus excretion. Supporting evidence are the virus RNA and virus antigen expression results of respective organs and virus RNA loads in swabs of oropharynx, cloaca, and preen gland. We tested for environmental virus contamination, and detected very low viral loads (Ct-value 39) in one of five samples of water surrounding the southernmost island of the archipelago, while all three other samples taken form shallow wetlands on this island tested negative, possibly due to the sun’s UV-light and/or heating up [27]. For the respiratory tract, virus produced in lung and airways could be excreted via airways to nasal and oral cavities; for the digestive tract, virus produced in the liver (excretion via bile duct and intestine) and pancreas (via pancreatic duct and intestine) could be excreted via the cloaca; for the urinary tract, virus produced in the kidney could be excreted via ureters and cloaca; for the reproductive tract, virus produced in the gonads could be excreted via ductus deferens (or oviduct in the tern) to the cloaca; and for the preen gland, virus produced in the glandular acini could be excreted via the primary duct. Previously, Ct-values ranging from 22 to 29 for oropharyngeal swabs and from 19 to 31 for cloacal swabs indicative of virus RNA excretion were detected in four black-headed gulls found dead with HPAI H5N1 virus infection in England in 2022 [28]. Black-headed gulls inoculated experimentally with HPAI H5N1 Gs/GD virus (A/turkey/Turkey/1/2005) [21] showed virus RNA excretion detected in oropharyngeal and cloacal swabs from 1 day post inoculation until the end of experiment at day 12, whereas infectious virus was detected until day 7 with consistently higher loads and titres in the oropharynx than in the cloaca. These different sites of virus production and potential sites of virus excretion contrast with findings of natural LPAI H16N3 or H13N8 virus infection in black-headed gulls, where the site of virus production was the intestine and virus was excreted mainly via the cloaca [20].

The findings of the common tern were comparable to the findings of the black-headed gulls, indicating similar pathology and pathogenesis. However, virus antigen expression in hepatocytes and in epithelial cells of the ovary and in neurons of the intestinal nerve plexus of Meissner was exclusively observed in the tern and not in the gulls. Even so, in absence of other such field pathology studies in common terns, the significance of our results in one tern is limited.

In summary, our results indicate that the pathogenesis of HPAI H5N1 clade 2.3.4.4b genotype BB virus infection in adult breeding black-headed gulls was defined by an extraordinary fast development of multisystemic fatal disease. This was evidenced by acute necrosis without inflammation and extensive extra-respiratory organ involvement, including critically affected organs, brain and pancreas, resulting in severe neurological signs and rapid death. The main difference with experimental infections of black-headed gulls with an older strain of HPAI H5N1 virus [21] was the more acute disease. Additionally, the preen gland must be considered a possible route of HPAI H5N1 virus excretion in birds, besides oropharyngeal and cloacal excretion.

The implications of this study in combination with other epidemiological knowledge of genotype BB virus in black-headed gulls [11] may be that this genotype or its descendent viruses could persist in future black-headed gull populations, possibly in the same way as LPAI viruses H13 and H16 appear to persist [36], and cause annual epidemics mainly restricted to fledgling black-headed gulls on colony sites [37], indicative of a degree of population immunity [38]. Persistence within the population may be enhanced by virus evolution towards decreased virulence, following the trade-off hypothesis or intermediate virulence hypothesis [39], resulting in more efficient transmission by not killing its host prematurely. Continued HPAI-surveillance in wild birds is essential to follow the evolution of HPAI H5N1 Gs/GD virus in wild birds, and to fill other knowledge gaps that may help to mitigate future HPAI outbreaks [40, 41] and to protect wildlife, domestic animals, and human health according to the One Health approach [42].

## Supplementary Information

**Additional file 1. Map of the island group that forms the small man-made archipelago "de Kreupel" (geographical coordinates 52°47'56.0"N latitude, 5°13'32.1"E longitude) in The Netherlands that harbours breeding colonies of several bird species including black-headed gulls (*Chroicocephalus ridibundus*) and common terns (*Sterna hirundo*).** The letters G denote the locations where black-headed gulls were collected, T denotes the location of the common tern collected, and the letters W on the southernmost island denote water sampling locations; W1-3, 7 and 8 from surrounding waters, and W4-6 from shallow rainwater wetlands or pools on the island. Many gulls and terns, dead or alive, were observed on this island also. Sample W3 was the only water sample that tested (weakly) positive for influenza A(H5) virus RNA.

**Additional file 2. Photographs of water sampling locations from the southernmost island of the archipelago "de Kreupel"**. **A**, sample location (W3) of water that was taken from shaded areas in between the rocks, in the foreground of the photograph, that delineate the island, and it was the only sample from a total of eight water samples from different locations on, and surrounding, that island that tested weakly positive for influenza A(H5) virus RNA. **B**, sample location (W6) of one of three small shallow sunlight-exposed rainwater pools on the island that tested virus RNA negative. Note bird feathers and numerous bird footprints surrounding the pool.

## Data Availability

Genome sequences were deposited and available from GISAID database [25] with accession numbers: EPI_ISL_19854913, EPI_ISL_19855567, and EPI_ISL_19857471.
