# Supplementary figures and images for: Pathology and virology of natural high pathogenicity avian influenza A(H5N1) Gs/GD genotype BB virus infection in wild black-headed gulls (Chroicocephalus ridibundus)
Source: Vet Res. 2025 Dec 29;56:234. doi: 10.1186/s13567-025-01666-x (PMC12752434; doi:10.1186/s13567-025-01666-x)

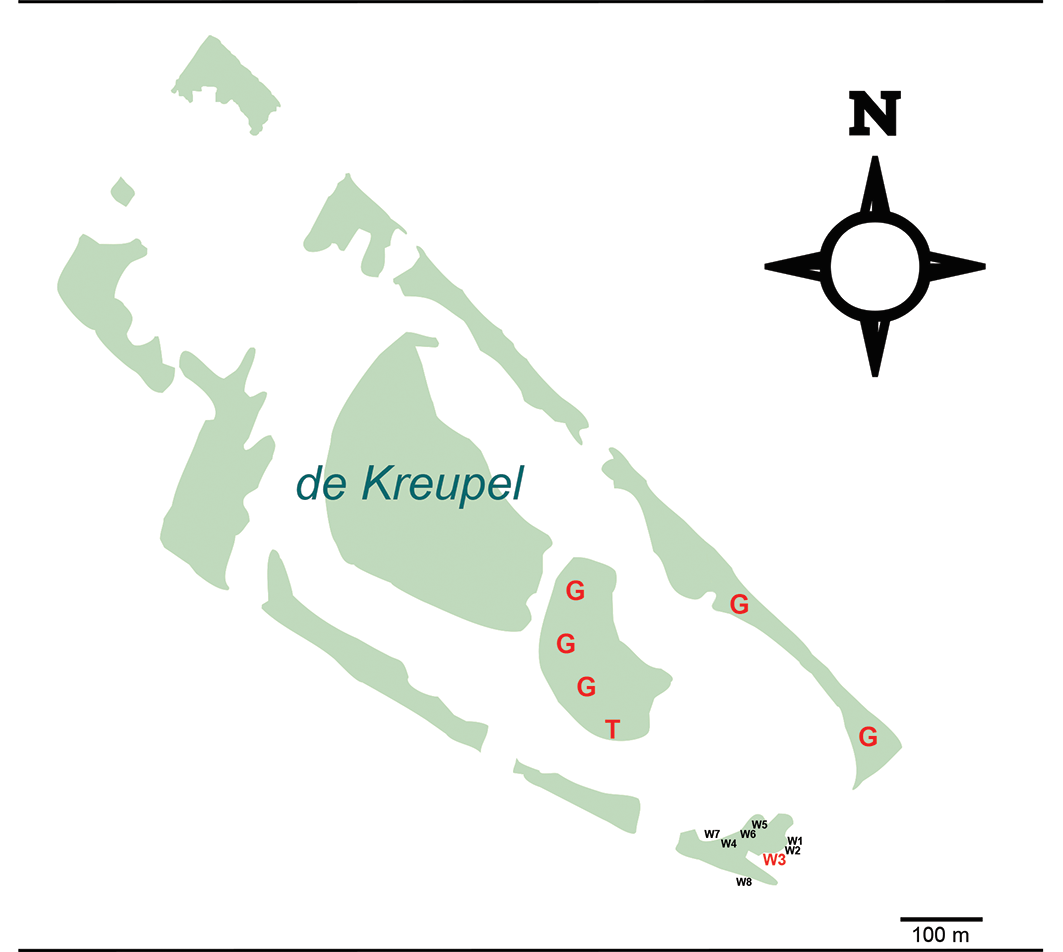

Supplement: Supplementary file 1 — Additional file 1. Map of the island group that forms the small man-made archipelago "de Kreupel" (geographical coordinates 52°47'56.0"N latitude, 5°13'32.1"E longitude) in The Netherlands that harbours breeding colonies of several bird species including black-headed gulls (Chroicocephalus ridibundus) and common terns (Sterna hirundo). The letters G denote the locations where black-headed gulls were collected, T denotes the location of the common tern collected, and the letters W on the southernmost island denote water sampling locations; W1-3, 7 and 8 from surrounding waters, and W4-6 from shallow rainwater wetlands or pools on the island. Many gulls and terns, dead or alive, were observed on this island also. Sample W3 was the only water sample that tested (weakly) positive for influenza A(H5) virus RNA. [file 13567_2025_1666_MOESM1_ESM.tif]

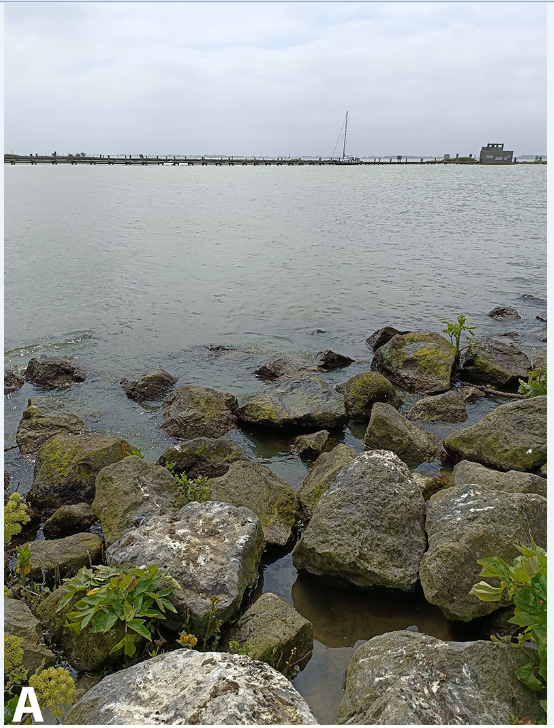


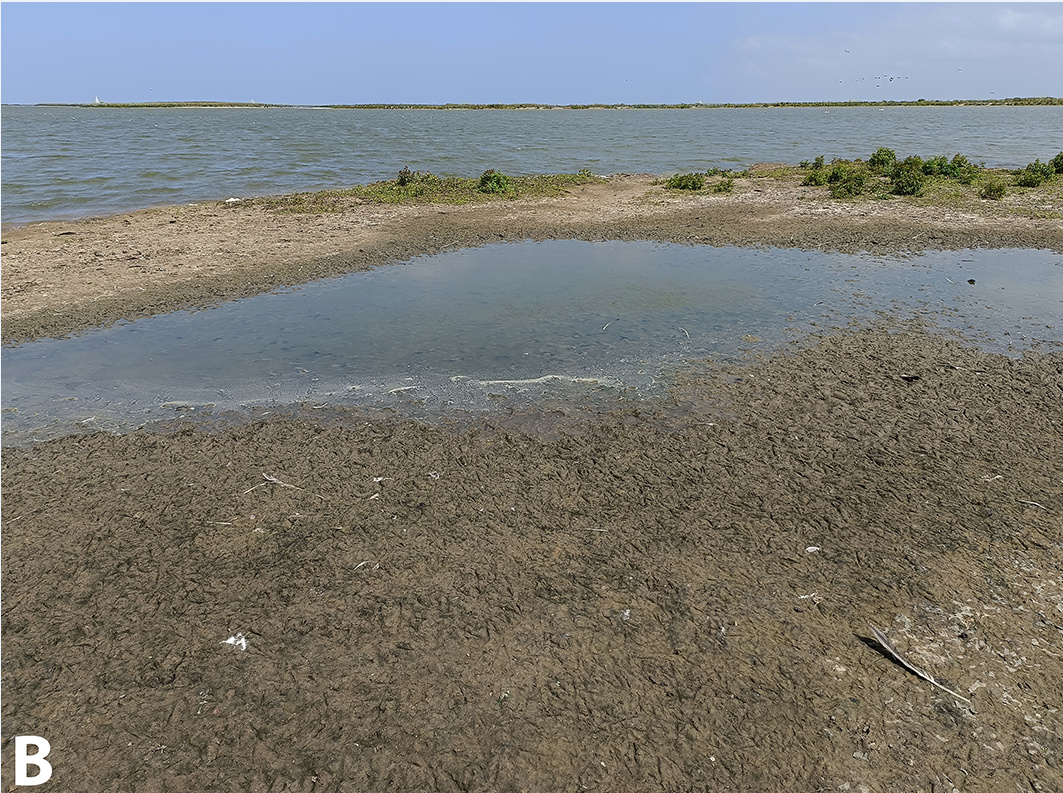

Supplement: Supplementary file 2 — Additional file 2. Photographs of water sampling locations from the southernmost island of the archipelago "de Kreupel". A, sample location (W3) of water that was taken from shaded areas in between the rocks, in the foreground of the photograph, that delineate the island, and it was the only sample from a total of eight water samples from different locations on, and surrounding, that island that tested weakly positive for influenza A(H5) virus RNA. B, sample location (W6) of one of three small shallow sunlight-exposed rainwater pools on the island that tested virus RNA negative. Note bird feathers and numerous bird footprints surrounding the pool. [file 13567_2025_1666_MOESM2_ESM.docx]
